# Analysis of neonatal brain lacking ATRX or MeCP2 reveals changes in nucleosome density, CTCF binding and chromatin looping

**DOI:** 10.1093/nar/gku564

**Published:** 2014-07-18

**Authors:** Kristin D. Kernohan, Douglas Vernimmen, Gregory B. Gloor, Nathalie G. Bérubé

**Affiliations:** 1 Department of Biochemistry, University of Western Ontario, London N6C 2V5, Canada; 2 Children's Health Research Institute, London, Canada; 3 Medical Research Council (MRC) Molecular Haematology Unit, Weatherall Institute of Molecular Medicine, University of Oxford, John Radcliffe Hospital, Oxford, UK; 4 Department of Paediatrics, University of Western Ontario, London N6C 2V5, Canada

## Abstract

ATRX and MeCP2 belong to an expanding group of chromatin-associated proteins implicated in human neurodevelopmental disorders, although their gene-regulatory activities are not fully resolved. Loss of ATRX prevents full repression of an imprinted gene network in the postnatal brain and in this study we address the mechanistic aspects of this regulation. We show that ATRX binds many imprinted domains individually but that transient co-localization between imprinted domains in the nuclei of neurons does not require ATRX. We demonstrate that MeCP2 is required for ATRX recruitment and that deficiency of either ATRX or MeCP2 causes decreased frequency of long-range chromatin interactions associated with altered nucleosome density at CTCF-binding sites and reduced CTCF occupancy. These findings indicate that MeCP2 and ATRX regulate gene expression at a subset of imprinted domains by maintaining a nucleosome configuration conducive to CTCF binding and to the maintenance of higher order chromatin structure.

## INTRODUCTION

ATRX is a chromatin remodeling protein with key functions in brain development and tumor suppression (1,2). Inherited mutations in the *ATRX* gene cause ATR-X syndrome, a severe intellectual disability disorder (3–5). The ATRX protein contains an N-terminal ADD domain that promotes heterochromatin binding and a C-terminal SWI/SNF domain that confers translocase and chromatin remodeling activities (6–12). It is targeted to specific genomic regions via other chromatin-bound proteins or specific histone modifications (10,13,14). Genome-wide studies have shown that ATRX is enriched at GC-rich and repetitive sequences including telomeres, many of which are predicted to form secondary deoxyribonucleic acid (DNA) structures called G-quadruplexes (15–17). ATRX has important functions in dividing cells, influencing processes like meiosis, mitosis, and DNA replication (17–21). Disruption of these activities may contribute to microcephaly and other developmental abnormalities associated with ATR-X syndrome.

ATRX deficiency alters gene transcript levels in both dividing and non-dividing cells (22). In the testes, ATRX was demonstrated to interact with the androgen receptor to regulate RhoX5 gene expression (23). However, the molecular mechanisms that underlie transcriptional control by ATRX in other tissues has not been addressed. ATRX interacts with several chromatin proteins including death domain associated protein (DAXX), heterochromatin associated protein 1 (HP1) and methyl-CpG binding protein 2 (MeCP2). MeCP2 targets ATRX to pericentromeric heterochromatin in the mouse central nervous system (13,24). In humans, mutations in the *MeCP2* gene cause Rett syndrome, a progressive neurodevelopmental disorder characterized by developmental regression beginning at 6–18 months of age (25). The timeline of Rett syndrome etiology has been attributed to the level of MeCP2 protein in the brain, which is low in the embryonic period and increases progressively after birth (25). We previously reported that ATRX and MeCP2 proteins bind the maternal allele of the *H19* imprinting control region (*H19* ICR), and that loss of ATRX prevented the repression of maternal *H19* transcription, as well as other co-regulated imprinted genes in the postnatal brain (24).

The present study addresses the molecular mechanism that could explain ATRX-mediated repression of imprinted genes in the neonatal brain. Our findings reveal that ATRX binds to differentially methylated regions (DMRs) including imprinting control regions (ICRs) at many imprinted domains. MeCP2 is required for ATRX binding, and loss of ATRX or MeCP2 results in increased nucleosome occupancy over CCCTC-binding factor (CTCF) binding sites. This failure to maintain an extended linker region necessary for stable CTCF binding explains reduced CTCF binding and the altered long-range chromatin interactions and gene expression at two imprinted regions, *H19/Igf2* and *Dlk1/Gtl2*. We propose a model where ATRX and MeCP2 cooperate to control nucleosome positioning at specific CTCF binding sites, thus enabling local chromatin looping configurations and repression of imprinted gene expression in the neonatal brain.

## MATERIALS AND METHODS

### Animal husbandry

The *Atrx* gene was conditionally deleted in the mouse forebrain as previously described (26). The *Atrx^loxP^* line was provided by D. Higgs and R. Gibbons (Weatherall Institute of Molecular Medicine, John Radcliffe Hospital, Oxford, UK). For allele-specific expression studies, pups were obtained by mating 129Sv female mice with *Mus musculus castaneus* males (CAST; The Jackson Laboratory). *MeCP2^null^* mice were generated by crossing *MeCP2^loxP^* females (Jackson Laboratories Stock #007177) with a ubiquitous *Cre* line driven by the EIIa promoter (Jackson Laboratories Stock #003724). Animal studies were conducted in compliance with the regulations of The Animals for Research Act of the province of Ontario, the guidelines of the Canadian Council on Animal Care, and the policies and procedures approved by the University of Western Ontario Council on Animal Care.

### Circular chromosome conformation capture (4C)

The 4C protocol was based on that previously reported by Gheldof *et al.* (27). Briefly, following 3C library preparation, DNA was digested with MseI (NEB) overnight at 37°C. The enzyme was deactivated for 25 min in 1.3% Sodium dodecyl sulfate (SDS) at 65°C and DNA recovered by standard phenol/chloroform extraction. Digestion efficiency was confirmed to be ≥96% by real-time PCR across five sites throughout the genome. DNA was resuspended in 7 ml ligation buffer with 50 U T4 DNA ligase (Roche Diagnostics) and 1 μM adenosine triphosphate (ATP) and incubated at 16°C for 5 days. DNA was purified by phenol/chloroform extraction and amplified with the Expand Long Template PCR system (Roche Diagnostics) and site-specific primers. PCR products were resolved on a 1% agarose gel, and extracted in three aliquots using a QIAquick gel extraction kit (Qiagen); undigested band, <230 and >230 bp. The >230 bp fraction was sheared enzymatically using the Ion Shear Plus Reagents, and then combined with the <230 bp fraction and 1/6th of the undigested self-ligation fragment before barcoding with the Ion Xpress Barcode Adapters 1–16 kit. Sequencing was performed using the Ion Torrent Personal Genome Machine (Life Technologies) with 318 chips and 200 bp sequencing chemistry according to manufacturer's protocols.

Ion Torrent sequence reads were aligned by TMAP Suite 3.2.1. A library of mouse genome EcoRI fragments was generated and the number of unique reads that mapped to each interval calculated. Because the ratio of reads in any interval to the total was very small, standard statistical techniques were used to construct a robust estimator of the underlying proportions (28). Specifically, underlying proportions were estimated using a multinomial-Poisson model in a Bayesian context using a minimally-informative reference prior (29,30). Because fold-change is the usual measure of effect-size, all expectations were taken with respect to log_2_-proportions (31). For compatibility with the UCSC genome browser, the expected log_2_-proportions were mapped back to linear-space and multiplied by an arbitrary integer-scaling factor. Strongly positive interactions are defined by the top 10% of interacting sites. 4C data sets have been deposited in GEO DataSets.

### Chromosome conformation capture (3C)

3C libraries were prepared essentially as previously described (32), with the same controls. Samples that lacked either the EcoRI or T4 DNA ligase enzymes were prepared in parallel. Digestion efficiency was confirmed to be ≥96% by real-time PCR across five sites spanning the *H19/Igf2* domain. Library amplification and quantification was conducted as described previously (32), with the same controls. Briefly, PCR reactions, primers and probes were optimized on a library of randomly ligated BAC DNA containing the *H19/Igf2* domain and XPB. All primer combinations amplified in linear correlation with the amount of BAC DNA and within 2 Cts. 3C data was corrected to primer efficiency and calculated relative to XPB/ERCC3 amplification (33). 3C templates obtained from P0.5 ATRX-null and littermate control forebrains were amplified in duplicate with Taqman Universal PCR Master Mix (Applied Biosystems) on a Chromo-4 thermocycler (BioRad) as per manufacturer's instructions. A negative bait site located approximately 100 kb downstream of *H19* was also used, to confirm specificity of ICR interactions.

### 3D DNA FISH and immunofluorescence microscopy

Neonatal brains were fixed overnight in 4% paraformaldehyde (Sigma-Aldrich), equilibrated in 30% sucrose–phosphate buffered saline (PBS), frozen in O.C.T. (Tissue Tek) and sectioned at 8 μm. Antigen retrieval was performed using 0.3% sodium citrate (Sigma-Aldrich) for 1 h. Slides were dehydrated in an ethanol series of 70% for 2 min, 90% for 2 min, and 100% for 5 min, followed by denaturation in 70% formamide/2× SSC for 5 min at 65°C. Slides were again dehydrated as described above and then incubated with 0.05 μg of DIG and/or biotin-labeled probe/hybridization buffer [83% formamide (Sigma-Aldrich), 3.3× SSC (Sigma-Aldrich), 0.02 μM dextran sulfate and 30 μg salmon sperm DNA (Sigma-Aldrich)] overnight at 37°C in a humidified chamber. Probes were prepared by nick translation of BAC DNA (*H19/Igf2*:RP23-50N22, *Gapdh*:RP23-319C23, *Peg10/Sgce*: RP23-327D3, *Dcn*:RP23228L10, *Slc38a4*:RP23-304B5, *Grb10*:RP23-298L21, *Dlk1:*RP23-385B6, *Zac1*:RP23-259L24, *Mest*:RP23-269K7) using the Biotin and DIG-Nick Translation Kits (Roche Diagnostics) and purified using the High Pure PCR Product Purification Kit (Roche Diagnostics) as per manufacturer's instructions. Slides were washed in 50% formamide/2× SSC for 2× 5 min and 2× SSC for 2× 5 min. Sections were incubated with the primary antibody for 1 h at room temperature, washed for 15 min in PBS and incubated with the secondary antibody for 1 h. Sections were counterstained with 4',6-diamidino-2-phenylindole (DAPI) (Sigma-Aldrich; D9542) and mounted in Slowfade Gold Antifade Reagent (Invitrogen). The primary antibodies used were as follows: anti-ATRX H-300 (1:250; Santa Cruz Biotechnology), anti-DIG (1:100; Roche Diagnostics) and anti-Biotin (1:500; Abcam). Secondary antibodies used were as follows: goat anti-rabbit Alexa 594 (1:800; Invitrogen) and goat anti-mouse Alexa 488 (1:800; Invitrogen). Images were taken at 0.3 μm intervals across the 8 μm section using the Olympus FV1000 confocal microscope and FV10-ASW 2.1 image acquisition software (Olympus). Volocity software (PerkinElmer) was used to compile 3D images and distances were measured using Volocity 3D measurement tools. For co-localization analysis, FISH signals with a center-to-center distance of <1 μm were considered to be interacting (34).

### ChIP analysis

Chromatin immunoprecipitation (ChIP) was conducted as previously described (24) with anti-ATRX (H300; Santa Cruz Catalog #sc-15408), anti-CTCF (Cell Signaling Catalog #2899), anti-H3.3 (Millipore Catalog #17-10245) and anti-H2A (Cell Signaling Catalog #2578). DNA–antigen complexes were retrieved by incubation with protein A agarose beads (Cell Signaling). Quantification was conducted as previously described (24).

### ChIP-sequencing analysis

Raw sequencing data for ATRX embryonic stem cell ChIP-sequencing was downloaded from the NCBI Sequence Read Archive (accession number: GSE22162), and aligned to the mouse genome using Bowtie version 0.12.8 in the −*n* alignment mode. During alignment duplicate sequences were removed, up to three mismatches were allowed, and reads that aligned to more than one location were discarded. Genome-wide data tracks were generated using custom Perl scripts to extend reads to their fragment lengths and normalized to 20 million reads. Data was visualized in the UCSC Genome Browser.

### Nucleosome density analysis

Neonatal mouse forebrain was dissected, rinsed in 37°C Dulbecco's modified Eagle's medium (DMEM) (Sigma-Aldrich) and passed through a 70-μm cell strainer (BD Falcon) to ensure single cell suspension. The cell suspension was incubated at 37°C for 30 min to equilibrate. Cells were fixed in 1% formaldehyde (Sigma-Aldrich) for 5 min and rinsed three times with cold PBS containing protease inhibitors (Roche Diagnostics). Cells were resuspended in lysis buffer [0.34 M sucrose, 60 mM KCl, 15 mM Tris–HCl, 15 mM NaCl, 0.5% NP-40 and 1× protease inhibitors (Sigma-Aldrich)] and flash-frozen and thawed three times, nuclei were centrifuged and resuspended in micrococcal nuclease digestion buffer (NEB). Micrococcal nuclease (2 U; NEB) was added and incubated at 37°C for 5 min, then quenched with EDTA. Cells were lysed with 1% SDS and cross-links reversed by incubation at 65°C for 5 h, followed by RNAse and PK digestion and phenol/chloroform extraction. DNA was subsequently digested with McrBC enzyme (NEB) for 2 h at 37°C. DNA was amplified in duplicate with iQ™ SYBR^®^ Green master mix (BioRad) on a Chromo-4 thermocycler (MJ Research) using the following conditions: 35 cycles of 95°C for 30 s, 57.5°C for 30 s, and 72°C for 1 min. Quantification was achieved using the Ct method of quantification and normalized to amplification of *Gapdh* and *Beta-actin.*

## RESULTS

### ATRX does not influence the co-localization of imprinted domains in the nuclei of neurons

**Figure 1.**
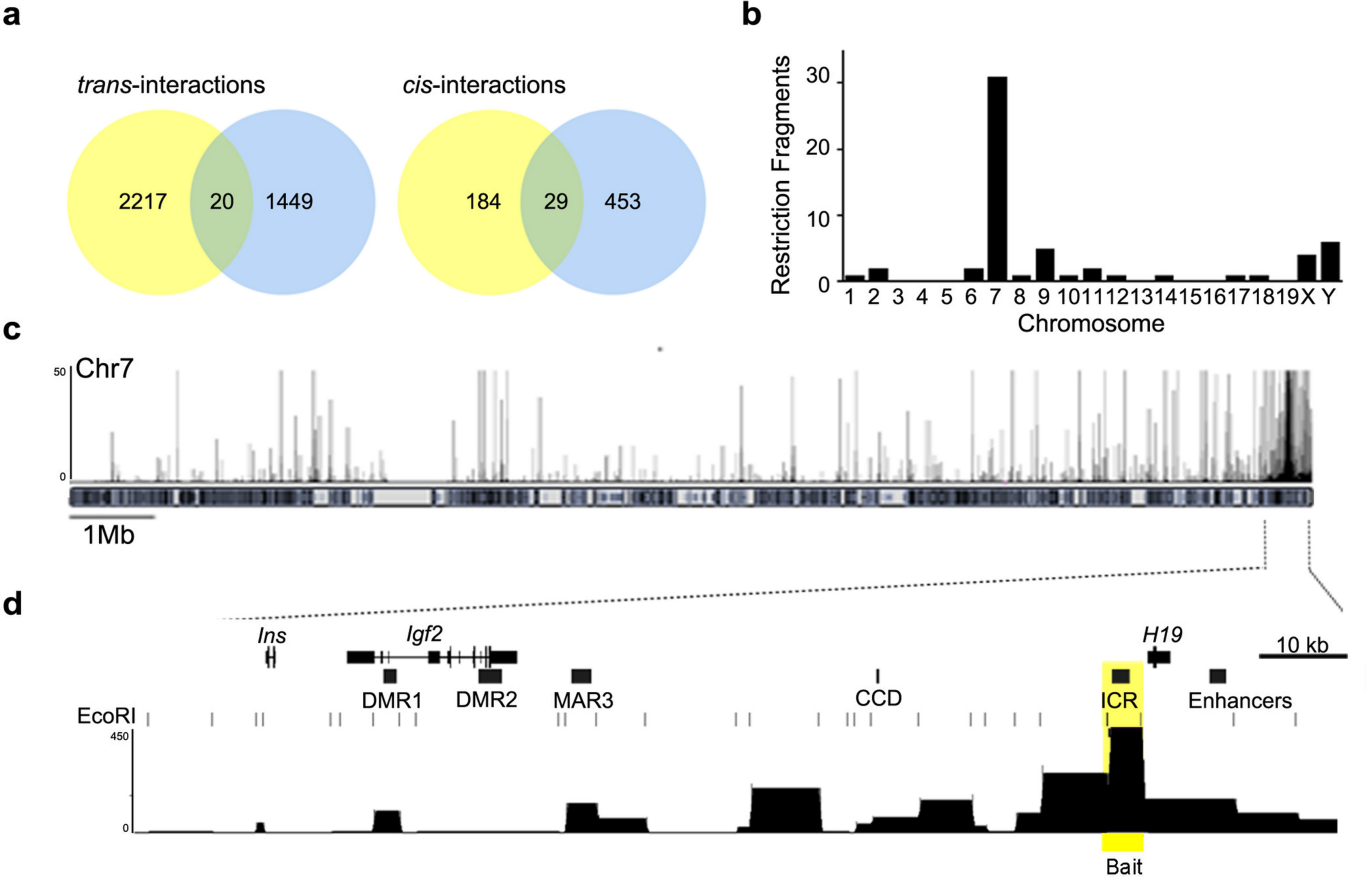
4C-sequencing analysis of chromosome interactions of the *H19* ICR bait sequence in neonatal mouse forebrain. (**a**) 3C libraries were generated from neonatal forebrain utilizing EcoRI, then re-digested with MseI and self-ligated to form circular 3C recombined molecules. The samples were then amplified with primers directed from the *H19* ICR ‘bait sequence’ across the interacting fragments and sequenced. Venn diagrams show the number of common sequences between 4C-seq biological replicates. Interactions of the *H19* ICR *in trans* are represented on the left and interactions *in cis* are represented on the right. (**b**) Analysis of genomic distribution of *H19* ICR interacting fragments on each chromosome reveals that the majority of reproducible interactions occur within chromosome 7 while *trans* interactions are distributed across the genome. (**c**) Representative 4C interaction profile across chromosome 7 (top) and the *H19/Igf2* imprinted domain (bottom). Local interactions are observe with the *Igf2* DMR1, MAR3, CCD and downstream enhancers. The 4C-seq data was aligned to an EcoRI digested genome and the *H19* ICR bait sequence is highlighted in yellow.

The *H19* ICR that regulates imprinting at the *H19/Igf2* domain was previously reported to interact with other imprinted domains located on other chromosomes (34,35) in a process requiring the CTCF chromatin organizer (34,35). Given that in the mouse brain, ATRX is required to achieve normal CTCF occupancy at the *H19* ICR (24), we proceeded to investigate whether ATRX co-regulates the imprinted gene network by promoting *inter*chromosomal interactions. We examined the pattern of chromatin fiber interactions mediated by the *H19* ICR using circular chromosome conformation capture with sequencing (4C-seq), a chromosome conformation capture (3C)-based technique that identifies genome-wide interactions *in vivo* from a single bait sequence (reviewed in (36)). 3C libraries were generated from neonatal (P0.5) forebrains with an EcoRI digestion. Following a second restriction digest with MseI, genomic fragments were self-ligated to form circular recombined molecules. PCR amplification was performed with primers directed from the *H19* ICR ‘bait sequence’ across interacting fragments and sequenced using Ion Torrent technology to provide an unbiased representation of genome-wide interactions. 4C analysis was conducted in two biological replicates and revealed that the *H19* ICR makes many contacts in the mouse forebrain. We identified 1951 and 2450 interactions in replicates 1 and 2, respectively. Among these sites we identified 20 *inter*chromosomal (*trans*) and 29 *intra*chromosomal (*cis*) regions that were common between the two replicates (Figure 1a and b, and Supplementary Table S1). The large majority of *intra*chromosomal contact sites occur within a 1 Mb distance from the *H19* ICR (28/29 sites), with 11 interactions occurring within 50 kb of the bait. Local contacts include the *Ins2*, *Igf2os* and *Igf2* genes, *Igf2* differentially methylated region 1 (DMR1), matrix-attachment region 3 (MAR3), centrally conserved domain (CCD) and the *H19* promoter (Figure 1c and d). These regions were previously reported to interact with the *H19* ICR in other cell types, validating our approach and confirming that very similar chromatin loops are formed in the neonatal forebrain (37–41). The imprinted genes regulated by ATRX were not included in the list of most robust interactions with the *H19* ICR bait. However, analysis of the data after binning sequences into 1 Mb intervals (Supplementary Figure S1a) showed interactions within 1 or 2 Mb of imprinted genes including *Grb10*, *Dlk1*, *Gtl2*, *Zac1* and *Mest*. Genomic intervals that include other imprinted gene network members also showed some enrichment, but failed to reach our threshold. The observation that interactions occur across a broad region surrounding imprinted genes, as opposed to specific restriction fragments, might reflect the dynamic nature of these interactions and transient co-localization of this network of imprinted genes.

Given that 4C data sets represent a snapshot of the combined chromatin interactions of millions of cells, we also examined the localization of *H19/Igf2* and other imprinted domains in single brain cells *in vivo*. 3D-DNA fluorescent in-situ hybridization (FISH) of neonatal forebrain cryosections showed that imprinted genes exhibiting increased expression in the ATRX-null forebrain (*Slc38a4*, *Grb10*, *Dlk1*, *Dcn*, *Zac1*, *Mest* and *Peg10/Sgce)* co-localize with *H19/Igf2* in neocortical cells with the exception of *Peg10/Sgce* (Supplementary Figure S1c and d). The co-localization frequencies are characteristic of transient events and are consistent with previously reported co-localization data (34). Importantly, no significant differences in the frequency of co-localization were detected in the absence of ATRX by DNA-FISH (Supplementary Figure S1d). These results indicate that imprinted domains co-localize transiently in cells of the mouse forebrain, but that these interactions do not require the ATRX protein.

### ATRX is required to maintain long-range chromatin interactions at the *H19/Igf2* imprinted domain

**Figure 2.**
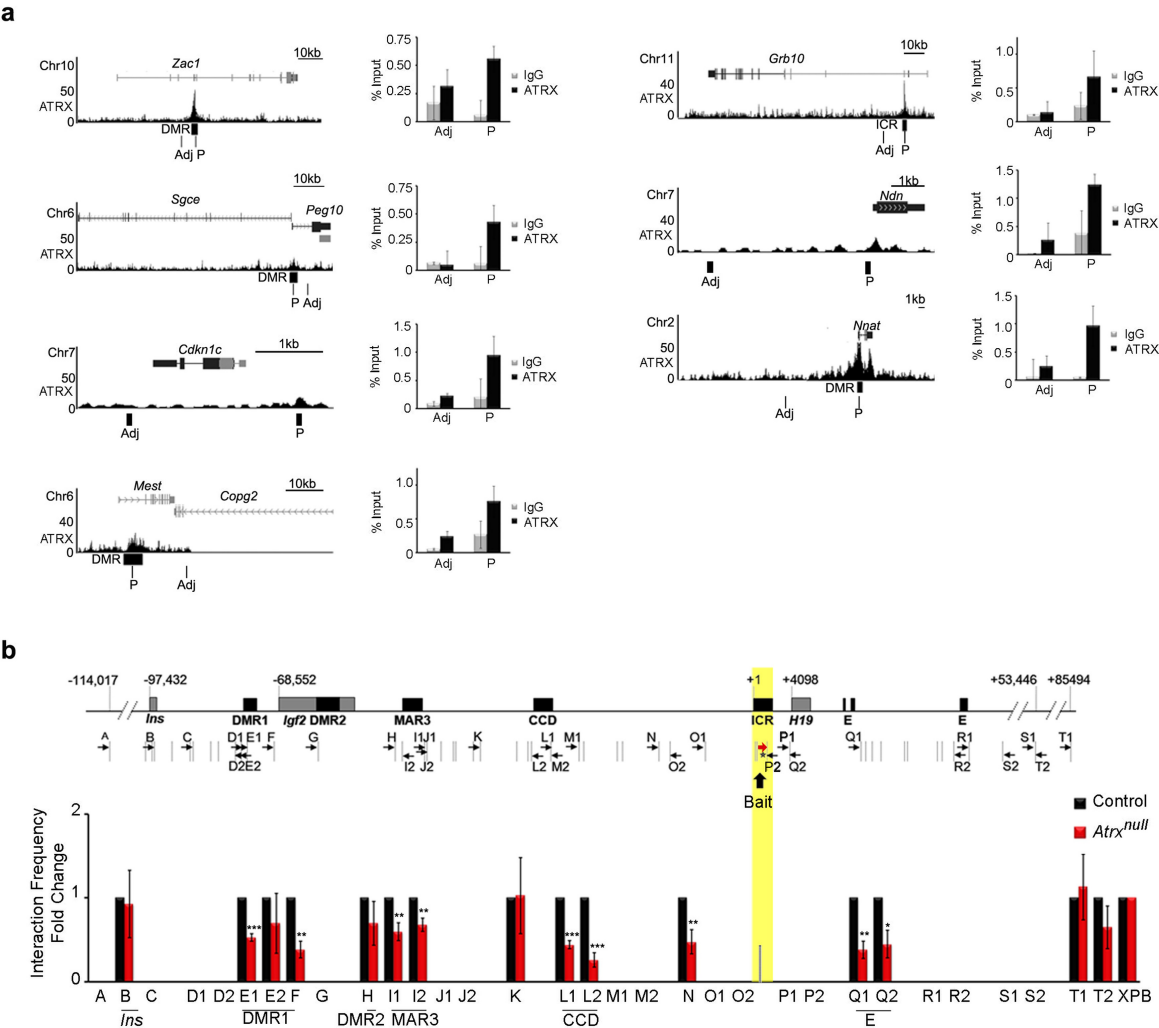
3C analysis of control and ATRX-deficient neonatal forebrain shows that ATRX is required for long-range chromosomal interactions mediated by the *H19* ICR. (**a**) Analysis of ATRX ChIP-sequencing data in mouse embryonic stem cells (15) shows ATRX occupancy at several imprinted domains (left panels, UCSC views). ATRX enrichment at these sites in neonatal mouse forebrain was confirmed by ChIP, as shown in the graphs on the left (*n* = 3, error bars represent SEM, p = peak, adj = adjacent). (**b**) Schematic representation of the *H19/Igf2* genomic region, the position of EcoRI sites (gray vertical lines) and the primers used for 3C analysis (black arrows). Gray boxes represent the position of genes and black boxes demarcate regulatory elements. Numbers indicate the relative nucleotide position from the start of the *H19* ICR. The *H19* ICR bait sequence is highlighted in yellow. 3C analysis was performed with the *H19* ICR bait and primers across the *H19/Igf2* domain in control and ATRX-null forebrains (*n* = 5 littermate pairs) and was quantified by PCR with a forward primer (red arrow), Taqman probe to the *H19* ICR (asterisk), and reverse primers. Graphed data represents the mean fold change of interaction frequencies, and error bars depict SEM. A two-tailed *t*-test was used to assess significance. *P < 0.05, **P < 0.01, ***P < 0.0001.

In addition to the *H19* ICR, ATRX binds a DMR found in the *Gtl2/Dlk1* imprinted region (24), and we wondered whether ATRX might directly bind each affected imprinted domain. To address this question, we analyzed ATRX ChIP-sequencing data previously obtained from embryonic stem cells (15). Analysis of the data revealed that ATRX indeed binds to many imprinted domains and that several binding sites overlap known DMRs and ICRs (Figure 2a). We were able to confirm that ATRX occupies the same genomic sites in the mouse forebrain by ChIP qPCR at *Zac1*, *Sgce/Peg10*, *Cdkn1c*, *Mest*, *Grb10*, *Ndn* and *Nnat* (Figure 2a).

The localization of ATRX at individual imprinted domains suggested that it might act *in cis* to regulate gene expression, rather than promoting the spatial proximity of imprinted domains in the nucleus. Furthermore, reduced occupancy of CTCF at the *H19* ICR in the ATRX-null forebrain might cause changes in chromatin looping at the *H19/Igf2* domain, which is known to affect gene expression. To address this possibility, we used quantitative chromosome conformation capture (3C) analysis, with primer design guided by the results of the 4C experiment. The approach included an EcoRI primary digestion, which divides the 140 kb *H19/Igf2* region into 45 fragments stretching from the *Ins* gene to the *H19* enhancers (Supplementary Figure S2a). We designed a forward primer and Taqman probe to the *H19* ICR (EcoRI restriction fragment used as bait), and 32 reverse primers in other EcoRI fragments covering negative intergenic regions as well as key genomic elements identified by 4C. Confirmation of interaction frequencies at a subset of sites was obtained by designing a second primer at the other end of the restriction fragment. To verify that our 3C experiments yield reliable data, we first tested the approach using neonatal liver. The results were comparable to those obtained by Qiu *et al.* (42) and to the interaction profile that we next generated using neonatal forebrain (Supplementary Figure S2b). As a negative control, we also designed a bait sequence located 100 kb downstream of the *H19* ICR, and this bait did not interact across the *H19/Igf2* region (Supplementary Figure S2b). We observed minimal or no interaction with a number of other sites (A, C, D, G, J, M, N, O, P, R, S), substantiating the specificity of the identified interactions. We then repeated the 3C analysis in control and ATRX null neonatal forebrain tissue isolated from littermate-matched pups. In forebrains lacking ATRX protein, we detected fewer interactions across the *H19*/*Igf2* domain, with significant reductions specifically at the *Igf2* DMR1 (region E/F: *P* = 0.001 and 0.0003), MAR3 (region I: *P* = 0.0052 and 0.0038), CCD (region L: *P* = 0.0001 and 0.0001) and the endodermal enhancer (region Q: *P* = 0.0004 and 0.0105) (Figure 2b). Conversely, interactions with *Ins2* (region B), the intergenic site between MAR3 and the CCD (region K) and the region downstream of the *H19* enhancers (region T) were not affected (Figure 2b). Importantly, the observed changes in interaction frequencies parallel the maternal-specific effects caused by CTCF deficiency in other cell types (40). For a subset of samples, expression analysis was performed in tandem with the 3C experiments to verify that gene expression and chromatin interaction frequencies were altered in the same samples (Supplementary Figure S2c and d).

### ATRX regulates nucleosome positioning at CTCF binding sites

**Figure 3.**
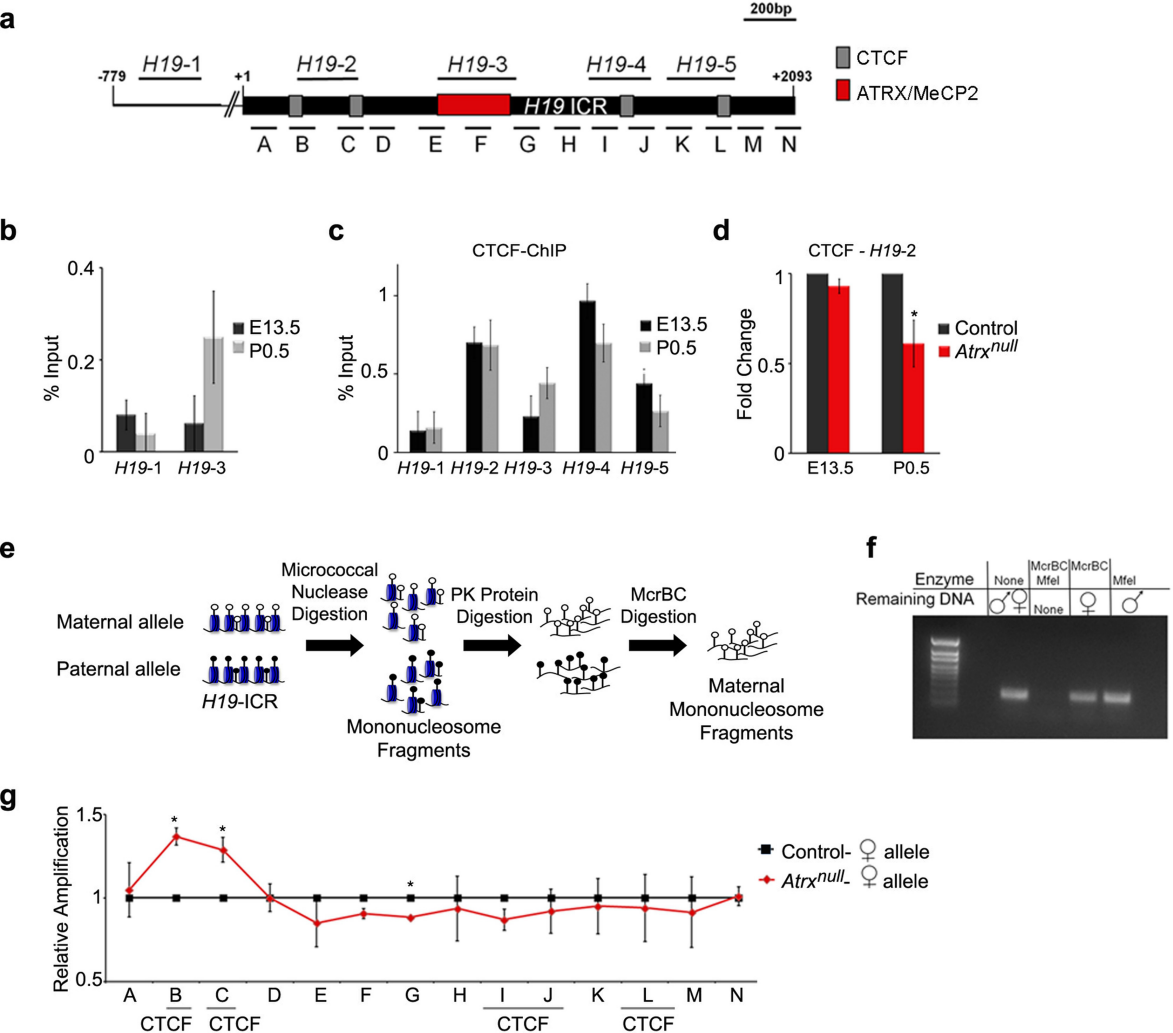
ATRX regulates nucleosome occupancy and CTCF binding at the *H19* ICR. (**a**) Schematic representation of the *H19* ICR and alignment of primers used for ChIP-qPCR (top) and nucleosome occupancy analysis (bottom). Gray boxes indicate the positions of CTCF binding sites and a red box marks the ATRX-binding site. Numbers indicate the relative position from the start of the *H19* ICR. (**b**) ChIP of ATRX in E13.5 and P0.5 forebrains shows binding of ATRX in the middle portion of the H19 ICR (H19-3) at P0.5, as observed previously, but not at E13.5, indicating that ATRX is recruited to the *H19* ICR in the late embryonic/neonatal brain. (**c**) CTCF ChIP at E13.5 and P0.5 shows binding of CTCF at both time points at previously identified areas of occupancy (H19-2 and H19-4). (**d**) ChIP of CTCF in control and ATRX-null forebrain tissue at E13.5 and P0.5 reveals that ATRX binding is required for CTCF binding at P0.5, but not at E13.5. Graphs in (b), (c) and (d) represent mean values, *n* = 3 for each and error bars depict SEM. (**e**) Diagram depicting the methodology used for allele-specific micrococcal nuclease digestion. Empty circles indicate unmethylated CpGs and black circles indicate methylated CpGs. (**f**) Validation of allele-specificity of nucleosome occupancy protocol, in which the methylated paternal *H19* ICR sequence is digested by McrBC. In F1 polymorphic 129Sv (maternal)/castaneous (paternal) forebrain samples, MfeI digests 129Sv maternal DNA and McrBC digests methylated paternal DNA. Following digestion, DNA was amplified using primers spanning the MfeI restriction site. (**g**) qPCR of micrococcal nuclease and McrBC-digested DNA reveals increased protection at the 5′ end of the maternal *H19* ICR in the ATRX-null samples. A significant increase in nucleosome occupancy was observed at regions B (*P* = 0.016) and C (*P* = 0.05) of the *H19* ICR and a significant decrease at adjacent site G (*P* = 0.001). Graph shows mean fold change and statistical analysis was performed by a two-tailed *t*-test (*n* = 3, errors bars depict SEM). *P < 0.05.

The repression of imprinted genes by ATRX is temporally regulated (24). To further explore this we first wanted to establish the state of ATRX and CTCF binding at the *H19* ICR before and after birth in the brain. ChIP analysis of ATRX shows that it does not bind to the *H19* ICR at E13.5, but does occupy this site at P0.5, indicating that ATRX is recruited to the *H19* ICR in the late gestational/neonatal period (Figure 3a and b). CTCF is bound to the ICR at E13.5 and P0.5 (Figure 3c) but in the ATRX-null samples, CTCF binding is undisturbed at E13.5, but significantly reduced at P0.5 (Figure 3d), corresponding to the recruitment of ATRX. This suggests that CTCF binding is independent of ATRX in the embryonic brain, but requires ATRX at a later stage. However, the mechanism by which ATRX regulates chromatin architecture and CTCF binding is still unclear and we hypothesized that ATRX stabilizes CTCF binding to chromatin. It was previously proposed that ATRX targets tandem repeat sequences that form G-quadruplexes, and that ATRX helps to deposit histone H3.3, at least at telomeres and pericentromeric heterochromatin (9,15,43). Based on these reports, we investigated whether these mechanisms contribute to ATRX-mediated CTCF occupancy at the *H19* ICR. Imprinted domains are enriched for tandem repeats, but analysis of the *H19/Igf2* region revealed that repeat sequences are located outside of the *H19* ICR and cannot explain ATRX targeting to this site (44). Additionally, the *H19* ICR sequence is not particularly GC-rich and is not predicted to form G-quadruplexes (45). Surprisingly, ChIP of H3.3 in neonatal forebrains showed that ATRX-deficiency induces a small increase in H3.3 within the *H19* ICR, in contrast to the decreased enrichment seen at ATRX-deficient telomeres (43) (Supplementary Figure S3a). However, a parallel ChIP of histone H2A yielded similar results, suggesting that elevated H3.3 ChIP signal at the ICR is not caused by increased H3.3 deposition, but rather might reflect differences in nucleosome occupancy (Supplementary Figure S3b).

**Figure 4.**
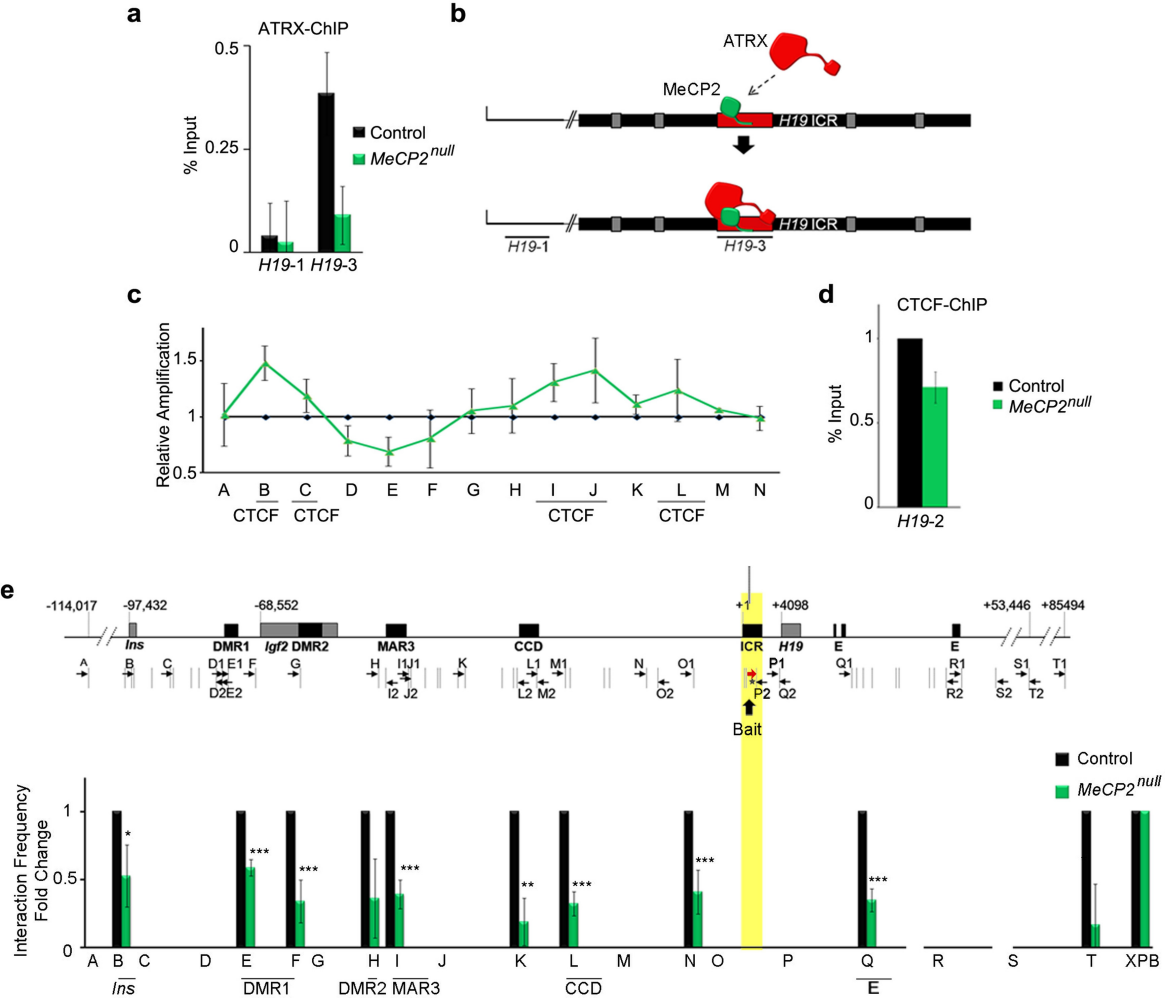
MeCP2 is required for ATRX and CTCF binding to the *H19* ICR and the long-range chromatin interactions across the *H19/Igf2* domain. (**a**) ATRX ChIP was performed in control and *MeCP2^null^* neonatal forebrain and shows that MeCP2 is required for ATRX occupancy at the *H19* ICR. The graph shows mean fold change value (*n* = 4) and error bars depict SEM. (**b**) Diagram of the *H19* ICR and location of the *H19-1* and *H19-3* primer pairs used in the ChIP-qPCR in (a). (**c**) Allelic nucleosome digestion assay of the *H19* ICR was performed in control and *MeCP2^null^* forebrains and reveals increased protection at the 5′ end of the maternal *H19* ICR in the *MeCP2^null^* neonatal forebrain. A significant increase in nucleosome occupancy was observed within regions B and I (*P* = 0.042) of the *H19* ICR and a significant decrease at site E (*P* = 0.05). Graphs depict mean fold change and statistical analysis was performed by a two-tailed *t*-test (*n* = 3, errors bars depict SEM). *P < 0.05. (**d**) CTCF ChIP at the 5′ end of the H19 ICR (*H19-2*) shows decreased CTCF occupancy at this site in the *MeCP2^null^* neonatal forebrain. The graph shows mean fold change value (*n* = 3) and error bar depicts SEM. **(e)** Schematic representation of the *H19/Igf2* genomic region, the position of *Eco*RI sites (gray vertical lines) and the primers used for 3C analysis (black arrows). Gray boxes represent the position of genes and black boxes demarcate regulatory elements. Numbers indicate the relative nucleotide position from the start of the *H19* ICR. The *H19* ICR bait sequence is highlighted in yellow. 3C analysis was performed with the *H19* ICR bait and primers across the *H19/Igf2* domain in control and *MeCP2^null^* forebrains (*n* = 3 littermate pairs) and was quantified by PCR with a forward primer (red arrow), Taqman probe to the *H19* ICR (asterisk), and reverse primers. Graphed data represents the mean fold change of interaction frequencies, and error bars depict SEM. A two-tailed *t*-test was used to assess significance. *P < 0.05, **P < 0.01, ***P < 0.0001.

CTCF binds within an extended linker region between nucleosomes (46,47) and *in vitro* studies showed that irregular placement of a nucleosome within a CTCF binding site prevents the association of CTCF with that region (48). We thus speculated that ATRX, using its ATP-dependent chromatin remodeling activities, might regulate the position of nucleosomes at the maternal *H19* ICR, perhaps creating a larger linker region to facilitate CTCF binding. Because ATRX and CTCF bind the maternal allele of the *H19* ICR, we devised a strategy to test allele-specific nucleosome occupancy. Chromatin from control and ATRX-null forebrains was digested with micrococcal nuclease and then with McrBC, an enzyme that degrades methylated DNA and should eliminate the highly methylated paternal *H19* ICR (Figure 3e). The allele-specificity of this assay was validated using brain samples obtained from 129Sv/CAST polymorphic mice that have sequence differences between the paternal and maternal alleles (Figure 3f). Using this approach, we were able to compare nucleosome protection of the maternal *H19* ICR in control and ATRX deficient neonatal forebrains. In the ATRX-null samples, we observed increased nucleosome protection in the region of the maternal ICR corresponding to the ATRX-dependent CTCF-bound area (primer pairs B and C, Figure 4g). In the absence of ATRX, abnormal nucleosome placement is predicted to impede CTCF binding, providing a mechanistic explanation for aberrant chromosomal looping and *H19/Igf2* gene expression.

### MeCP2 is required for ATRX recruitment and chromatin looping at *H19/Igf2*

One question remaining was the reason for ATRX recruitment to the *H19* ICR specifically after birth in the brain. MeCP2 was a prime candidate for promoting ATRX binding because it associates directly with the ATRX protein (13) and it co-localizes with ATRX at the *H19* ICR and *Gtl2* DMR in the neonatal brain (24). Furthermore, MeCP2 is required in neurons for the localization of ATRX at DAPI-rich heterochromatin bundles in the nucleus (49,50). To test whether MeCP2 is required for ATRX binding to the ICR, we performed ChIP for ATRX in control and MeCP2-null forebrain. Whereas ATRX was present at the *H19* ICR in control brains, it was not detected at this site in the absence of MeCP2 (Figure 4a and b), providing the first evidence that MeCP2 is required to recruit ATRX at specific genomic sites other than pericentromeric heterochromatin, at least at early stages of postnatal brain development.

If MeCP2 is in fact required for ATRX binding, we predicted that its absence should cause defects similar to those seen in the absence of ATRX. We conducted allele-specific nucleosome occupancy analysis in control and MeCP2-null forebrains and observed a similar pattern of altered DNA protection at sites B and C of the *H19* ICR, and interestingly found that nucleosome placement is also affected at the 3′ portion of the *H19* ICR (sites I and J) (Figure 4c). We also observed that CTCF binding is significantly reduced in the MeCP2-null neonatal forebrain (Figure 4d) at the *H19* ICR. Finally, 3C analysis detected a significant decrease in chromatin interactions mediated by the *H19* ICR in MeCP2-null forebrains. Loss of MeCP2 resulted in reduced interaction frequencies between the *H19* ICR (bait) and the *Igf2* DMR1, MAR3, CCD, as well as with the endodermal enhancer, similar to the results obtained in the ATRX-deficient brain (Figure 4e). Decreased interactions were also observed with *Ins2*, and the intergenic regions K and N (Figure 4e), showing that loss of MeCP2 affects interactions over a larger genomic region compared to loss of ATRX. These results show that MeCP2 can regulate chromatin structure at the *H19/Igf2* imprinted domains in the brain at an early perinatal period, and part of this regulation involves the recruitment of the ATRX chromatin remodeling protein to the *H19* ICR.

### ATRX and MeCP2 control nucleosome positioning and chromatin structure at the *Gtl2/Dlk1* imprinted domain

**Figure 5.**
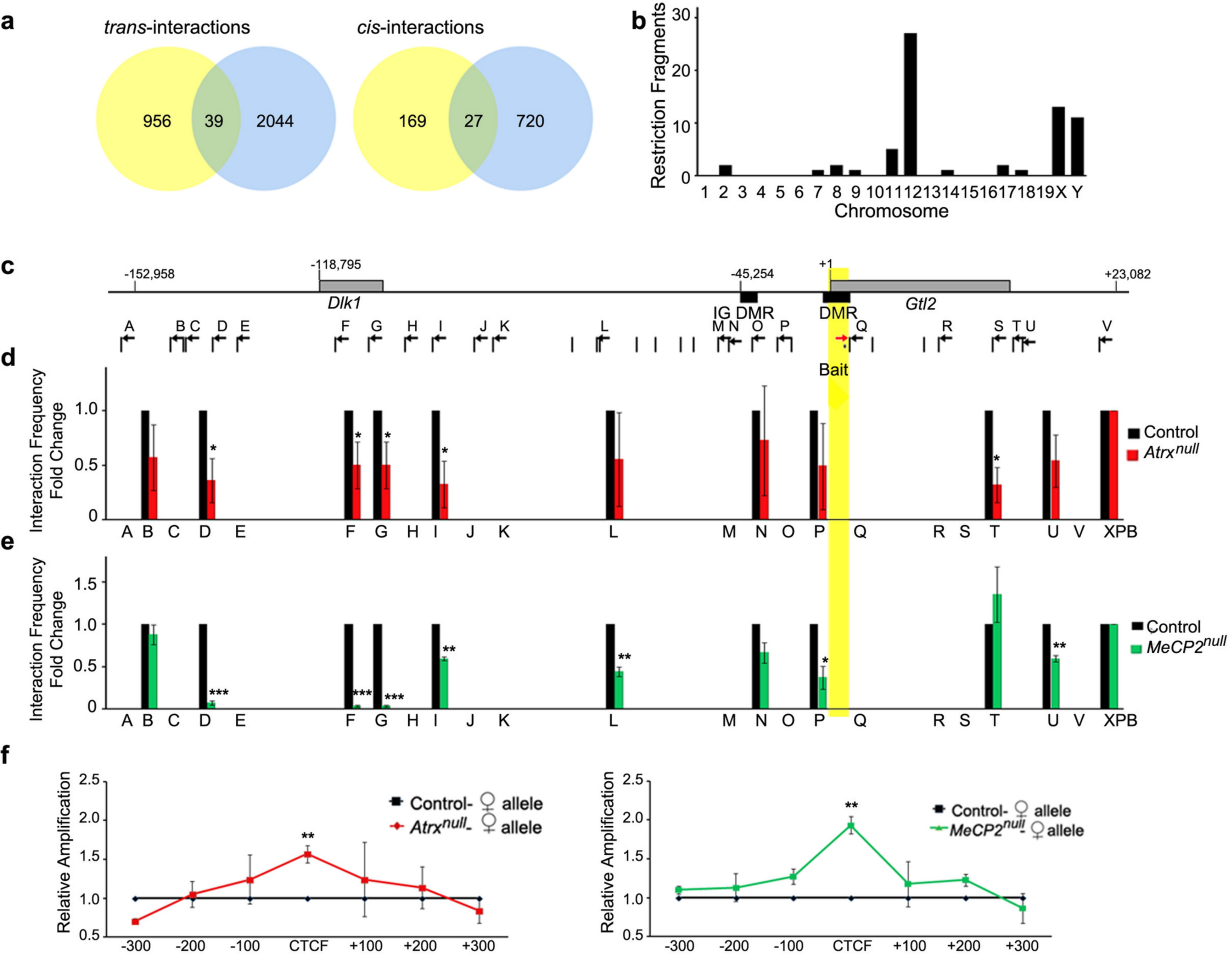
ATRX and MeCP2 regulate nucleosome positioning and long-range chromatin interactions mediated by the *Gtl2* DMR. (**a**) 4C-sequencing analysis using the *Gtl2* DMR as bait was performed in wild-type neonatal forebrains. Venn diagrams show the number of common sequences between 4C-seq biological replicates. Interactions of the *Gtl2* DMR *in trans* are represented on the left and interactions *in cis* are represented on the right. (**b**) Analysis of genomic distribution of *Gtl2* DMR-interacting fragments on each chromosome reveals that the majority of reproducible interactions occur within chromosome 12 while *trans* interactions are distributed across the genome, with a noted enrichment on the sex chromosomes. (**c**) Schematic representation of the *Gtl2/Dlk1* genomic region, the position of EcoRI sites (black vertical lines) and the primers used for 3C analysis (black arrows). Gray boxes represent the position of genes and black boxes demarcate regulatory elements. Numbers indicate the relative nucleotide position from the start of *Gtl2*. The *Gtl2* DMR bait sequence is highlighted in yellow. (**d**) For 3C analysis, DNA was digested with EcoRI, ligated and quantified by real-time PCR with a forward primer (red arrow) and Taqman probe to the *Gtl2* DMR (asterisk), and reverse primers (black arrows). Analysis was performed in control and ATRX-null or control and MeCP2-null neonatal forebrains (*n* = 3 littermate matched pairs each). A significant reduction in interaction frequency is observed at specific sites including the *Dlk1* gene and many intergenic regions. Graphed data represents the mean fold change, and error bars depict SEM. A two-tailed *t*-test was used to assess significance. *P < 0.05, **p < 0.01, ***p < 0.0001. (**e**) qPCR of micrococcal nuclease and McrBC digested DNA reveals increased DNA protection in the ATRX-null (left) MeCP2-null (right) forebrain overlapping the CTCF binding site in the *Gtl2* DMR. Graphs depict mean fold change and statistical analysis was performed by a two-tailed *t*-test (*n* = 3, errors bars depict SEM). **P < 0.01.

We next wanted to investigate whether this mechanism of gene regulation is restricted to the *H19* ICR or occurs at other imprinted genes. Given that ATRX localizes with MeCP2 at the *Gtl2* DMR within the *Gtl2*/*Dlk1* domain, and controls CTCF binding at this site (24), we chose this imprinted region to test whether the loss of ATRX or MeCP2 would have similar effects. We first designed a 4C-seq assay to identify interactions mediated by the *Gtl2* DMR. The approach involved an EcoRI primary digestion and a DpnII secondary digestion. 4C-seq libraries were prepared in two biological replicates and amplified with primers directed from the *Gtl2* DMR bait sequence across unknown interacting sites. We identified 1191 and 2830 interactions in replicates 1 and 2, respectively. Among these were 39 *inter*chromosomal and 27 *intra*chromosomal sites (Figure 5a and b, and Supplementary Table S2). We validated chromatin fiber interactions mediated by the *Gtl2* DMR by designing a forward primer and Taqman probe to the *Gtl2* DMR bait (Figure 5c). A previous study used 3C to identify interactions of the *Gtl2* intergenic DMR, and suggested that these sites could represent regulatory regions (51). We designed 22 reverse primers at sites identified in the 4C analysis, as well as potential regulatory elements and a number of randomly chosen fragments throughout the domain. The results demonstrate that the *Gtl2* DMR forms a number of specific contacts across this very large (∼1 Mb) imprinted domain, including the *Dlk1* gene (regions F and G), intergenic DMR (region N), and many intergenic sites (regions B, D, I, L, P, T and U) (Figure 5d and e). The loss of either ATRX or MeCP2 significantly reduced chromatin interaction frequencies at specific sites, including the *Dlk1* gene (region F: ATRX *P* = 0.05, MeCP2 *P* = 0.0001; region G: ATRX *P* = 0.05, MeCP2 *P* = 0.0011), and intergenic sites (region D: ATRX *P* = 0.0451, MeCP2 *P* = 0.0003; region I: ATRX *P* = 0.07, MeCP2 *P* = 0.043; region L: MeCP2 *P* = 0.0013; region T: ATRX *P* = 0.0.009; region U: MeCP2 *P* = 0.0057). Interactions with the intergenic DMR were not significantly affected by the loss of ATRX or MeCP2 (region N). Furthermore, allele-specific nucleosome analysis revealed that loss of MeCP2 or ATRX caused a significant increase in nucleosome occupancy at the *Gtl2* DMR CTCF biding site (Figure 5f), suggesting that abnormal nucleosome placement may impede CTCF at the *Gtl2* DMR. Together, these data suggest that MeCP2 recruits ATRX to control local nucleosome positioning, CTCF binding and chromatin architecture at multiple imprinted domains.

## DISCUSSION

ATRX and MeCP2 are key chromatin regulators implicated in ATR-X and Rett syndromes, respectively. Loss of function of ATRX or MeCP2 in the mouse brain causes alterations in gene expression (22,52); however, understanding the molecular mechanisms involved has been challenging. To address this question, we focused on imprinted genes, which we had previously demonstrated to be altered in the ATRX deficient brain. An emerging theory proposes that a subset of imprinted genes are jointly regulated in a cell-type specific network (24,34,35,53–55). In the nervous system, this sort of coordinated control of gene expression might be necessary during cellular differentiation and/or neuronal maturation, and could be facilitated by close subnuclear proximity or even direct allelic interactions. CTCF can mediate the co-localization of this gene network in spermatogonia, but not in liver and ES cells, suggesting that network control is cell-type specific (35). We now extend these studies and show that neuronal imprinted gene network (IGN) members indeed come into close proximity in neurons but that these *trans*chromosomal interactions occur independently of ATRX. Rather, we show that ATRX binds directly to each imprinted domain and mediates *intra*chromosomal interactions and controls gene expression at each domain in parallel. Interestingly, we find that *inter*chromosomal interactions do not occur with specific genomic elements, but are enriched in areas surrounding imprinted genes. This result suggests that these interactions are not functional, but rather consequences of co-localization within the nucleus, perhaps at transcription factories. A recent study reported that Hi-C analysis in single cells shows consistent local interactions between cells, but that *inter*chromosomal interactions are highly variable (56). As such, single cell analysis is necessary to provide further insights into *inter*chromosomal interactions of the *H19* ICR.

**Figure 6.**
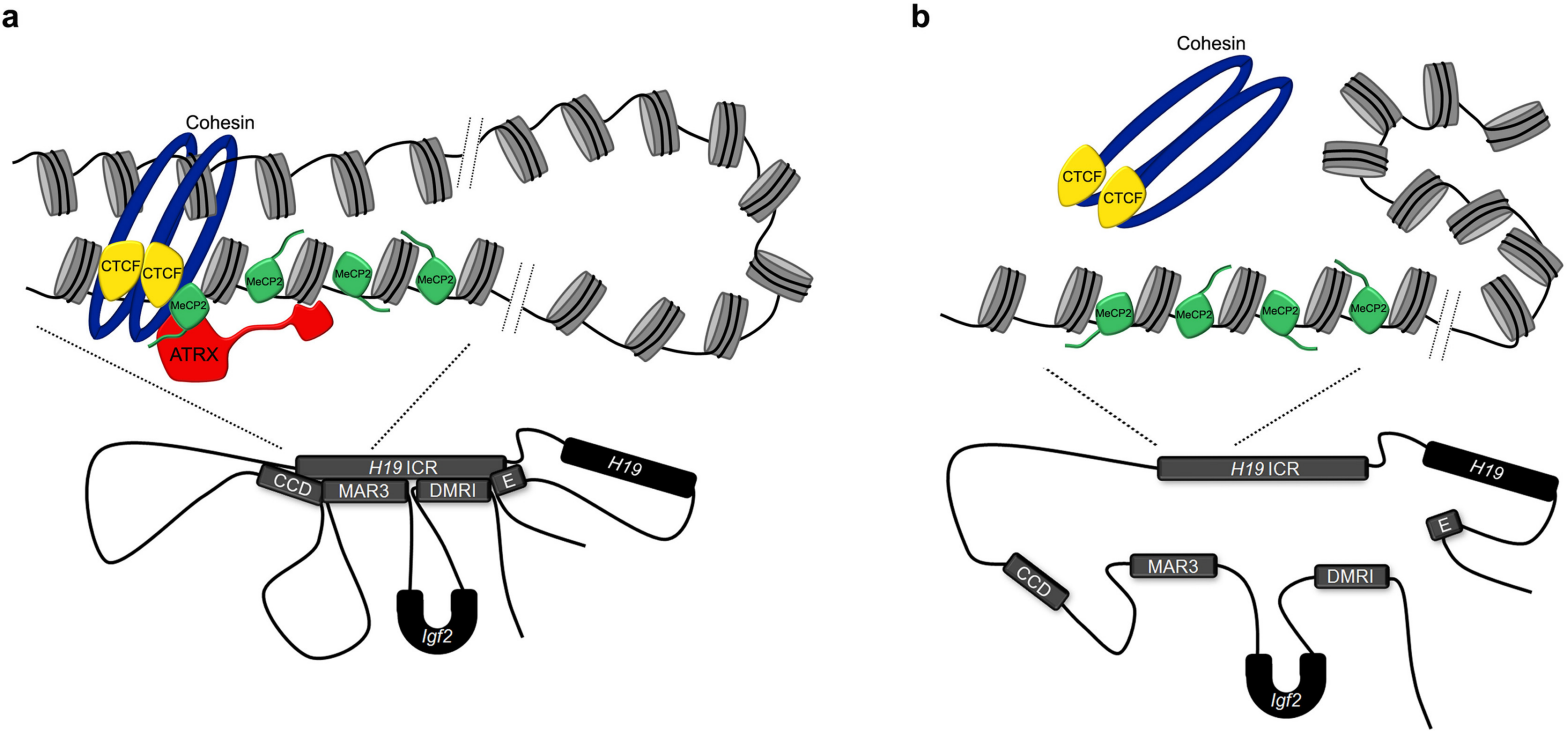
Model of ATRX and MeCP2 function. (**a**) In the wild-type brain, MeCP2 recruits ATRX to the maternal *H19* ICR in the late embryonic/neonatal period. ATRX translocates along the chromatin fiber and alters nucleosome positioning to generate an extended linker region and promote CTCF occupancy. CTCF then dictates *intra*chromosomal interactions. (**b**) In the absence of ATRX, increased nucleosome occupancy disrupts CTCF binding, leading to a loss of *intra*chromosomal interactions.

Our findings suggest a multi-step mechanism beginning with ATRX recruitment by MeCP2 in the post-neurogenic phase to repress gene expression (Figure 6). Upon binding to the DNA, ATRX repositions nucleosomes to create an extended linker region required for CTCF binding. In view of recent work showing that cohesin and CTCF enable higher-order chromatin looping within imprinted domains, and that ATRX alters CTCF and cohesin dynamics at these sites (24,37–40,57,58), a logical prediction was that the loss of ATRX would disrupt chromatin looping. We confirmed that this is indeed the case at *H19/Igf2* and *Gtl2/Dlk1*. At *H19/Igf2*, we report that loss of ATRX or MeCP2 resulted in reduced *H19* ICR chromatin interactions with the DMR1, MAR3, CCD and enhancer sequences. The disruptions in chromatin folding we observed (formation of an ICR-DMR1-MAR3 complex) parallel the maternal-specific effects following loss of CTCF (40). Unfortunately, it is not possible to confirm allele-specific effects by 3C in our system, as we are not able to obtain surviving polymorphic F1 ATRX-null mice (24). Nevertheless, a maternal allele-specific effect on chromatin interactions is corroborated by our previous study showing that ATRX, MeCP2, CTCF and cohesin bind specifically to the maternal *H19* ICR, and that loss of ATRX affects the levels of maternal, but not paternal *H19* transcripts in the neonatal forebrain (24). Furthermore, we show that ATRX or MeCP2 deficiency affects nucleosome occupancy on the maternal allele. It is unlikely that our observations are caused by altered cell populations, as these are minimal at birth. ATRX and MeCP2 bind directly to the *H19* ICR and *Gtl2* DMR, and at least at *H19*, the process of transcription or transcripts themselves do not affect chromatin looping (41). A reduction of chromatin contacts in the absence of ATRX or MeCP2 might increase gene expression in various ways. One scenario is that altered chromatin configurations expose the gene promoter to enhancers, or positions the gene at a greater distance from repressive elements. A full understanding of all genomic elements located within imprinted domains, and their role in silencing imprinted genes in the postnatal brain, will be required to understand the relationship between ATRX, MeCP2, chromatin structure and imprinted gene regulation.

ATRX is thought to bind G-quadruplex DNA and was proposed to resolve these structures to facilitate DNA replication and transcription (9,15,43). This mechanism has been proposed at telomeres (9,15,43) and at some specific genes (15). We now present a different mechanism of ATRX targeting and function that is independent of G-quadruplexes. We provide evidence that MeCP2 recruits ATRX to the *H19* ICR, where ATRX then modulates nucleosome occupancy within the 5′ region that overlaps two closely positioned CTCF-binding sites. Genome-wide studies have demonstrated that CTCF binds in an extended linker region. Binding of CTCF might require specialized enzymes, such as ATRX, to create the linker space required for stable CTCF binding. At the *H19* ICR, improper placement of a nucleosome within a CTCF binding site abrogates CTCF binding (48). Thus, correct positioning of nucleosomes within the ICR is required for CTCF to stably bind this site. It is also possible that altered nucleosome density is a consequence rather than the cause of loss of CTCF. At the moment, we favor a model where ATRX directly affects nucleosome density at the maternal ICR based on its demonstrated remodeling activities. ATRX may bind DNA adjacent to the CTCF binding sites via its ADD domain, which recognizes specific histone modifications. The SWI/SNF domain located at the end of a long flexible protein region is likely responsible for the redistribution of nucleosomes along the chromatin fiber (6,12). In the absence of ATRX, altered nucleosome distribution is predicted to induce CTCF eviction from the *H19* ICR. This mechanism of CTCF regulation is similar to that proposed at the chicken lysozyme locus (59), and may occur throughout the genome by ATRX and other site-specific chromatin remodelers. It is not yet clear whether this function of ATRX is limited to imprinted genes, or whether it occurs genome-wide. ATRX-mediated CTCF binding suggests that a developmental switch occurs at the *H19* ICR in the neonatal brain to elicit gene silencing. While we still lack a complete picture of the events at the *H19* ICR at this time, *H19* silencing on the maternal allele likely requires protein recruitment (including ATRX and MeCP2), epigenetic modifications and changes in long-range chromatin interactions.

In 2005, MeCP2 binding at the *Dlx5/Dlx6* imprinted domain was proposed to control chromatin looping and gene expression (60). However, uncertainty remained regarding this particular function of MeCP2 as the analysis was not quantitative, and MeCP2 regulation of *Dlx5* was later contested (61–63). We now provide evidence that the loss of MeCP2 diminishes chromatin interactions across the *H19/Igf2* and *Gtl2/Dlk1* imprinted domains. Compared to the results in the ATRX-null brain, the loss of MeCP2 has a more severe effect on interaction frequencies and affects more sites. One explanation for this discrepancy is that MeCP2 recruits other factors (in addition to ATRX) to regulate chromatin structure at these imprinted domains. Genome-wide ChIP studies have shown that MeCP2 binds across the genome in a histone-like manner and affects the expression of multiple genes (64). In light of our results, we predict that MeCP2 functions at many genomic sites to recruit specific chromatin remodeling proteins and control nucleosome positioning and chromatin architecture. Importantly, we demonstrate that MeCP2 regulates chromatin structure during early brain development, despite relatively low protein levels in the nucleus. It has been proposed that the role of MeCP2 in chromatin compaction would only become evident during the brain maturation phase when MeCP2 protein levels are much higher and when Rett syndrome-like phenotypes become evident. Our results counter this assumption, and suggest that expanding future studies to earlier developmental stages will help to fully elucidate the role of MeCP2 in neurodevelopment.

The failure to fully suppress the expression imprinted genes in the neonatal brain might negatively impact cognitive abilities, given that their misexpression is known to cause neurodevelopmental syndromes (reviewed in (65)). Moreover, the identification of CTCF as a key player in this model is particularly relevant given the recent identification of human *CTCF* mutations in individuals with intellectual disability (66). Continued studies of chromatin dynamics and imprinted gene regulation in the central nervous system could potentially lead to novel therapeutics for children with intellectual disabilities.

## Supplementary Material

SUPPLEMENTARY DATA

## ACCESSION NUMBER

GSE22162.
