# Supplementary material for: Analysis of neonatal brain lacking ATRX or MeCP2 reveals changes in nucleosome density, CTCF binding and chromatin looping
Source: Nucleic Acids Res. 2014 Jul 18;42(13):8356–68. doi: 10.1093/nar/gku564 (PMC4117782; doi:10.1093/nar/gku564)
Supplement: SUPPLEMENTARY DATA [file supp_42_13_8356__index.html]

Analysis of neonatal brain lacking ATRX or MeCP2 reveals changes in nucleosome density, CTCF binding and chromatin looping — Analysis of neonatal brain lacking ATRX or MeCP2 reveals changes in nucleosome density, CTCF binding and chromatin looping — SUPPLEMENTARY DATA 

# Analysis of neonatal brain lacking ATRX or MeCP2 reveals changes in nucleosome density, CTCF binding and chromatin looping

## SUPPLEMENTARY DATA

**Files in this Data Supplement:**

- Supplementary Figure
