# Supplementary material for: Analysis of neonatal brain lacking ATRX or MeCP2 reveals changes in nucleosome density, CTCF binding and chromatin looping
Source: Nucleic Acids Res. 2014 Jul 18;42(13):8356–68. doi: 10.1093/nar/gku564 (PMC4117782; doi:10.1093/nar/gku564)
Supplement: SUPPLEMENTARY DATA [file supp_gku564_nar-00453-m-2014-File008.pdf]

Supplementary Figure 1

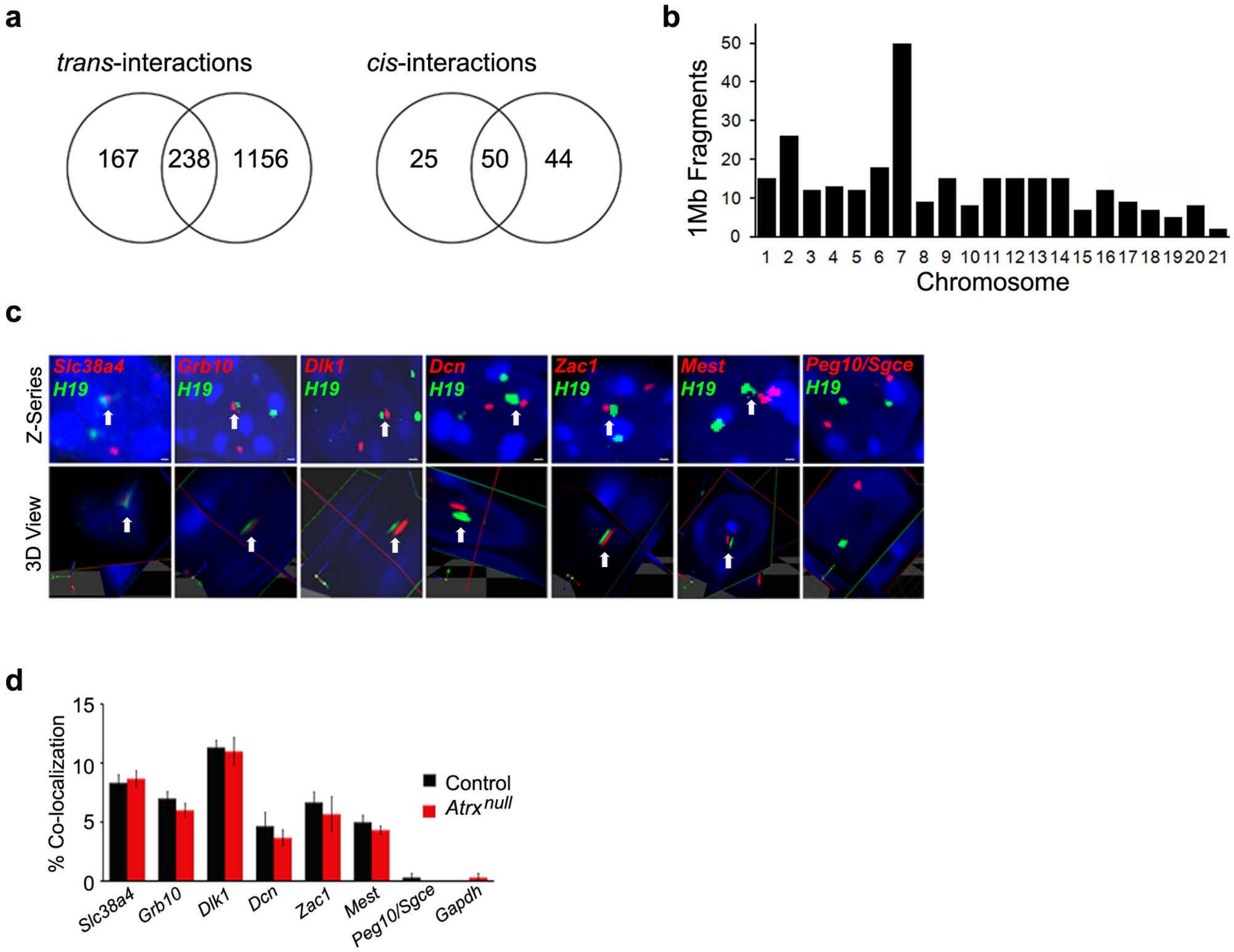

Supplementary Figure 2

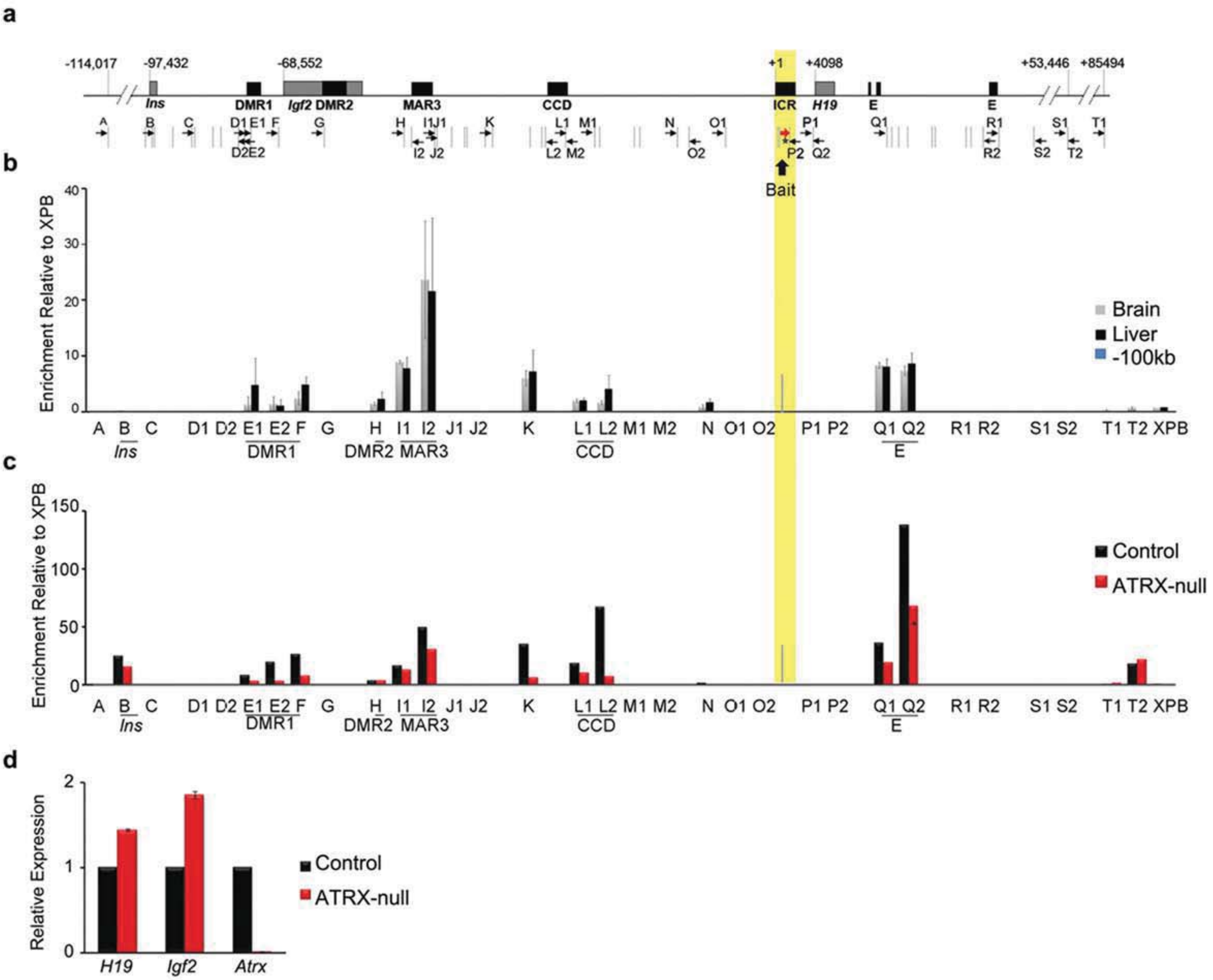

Supplementary Figure 3

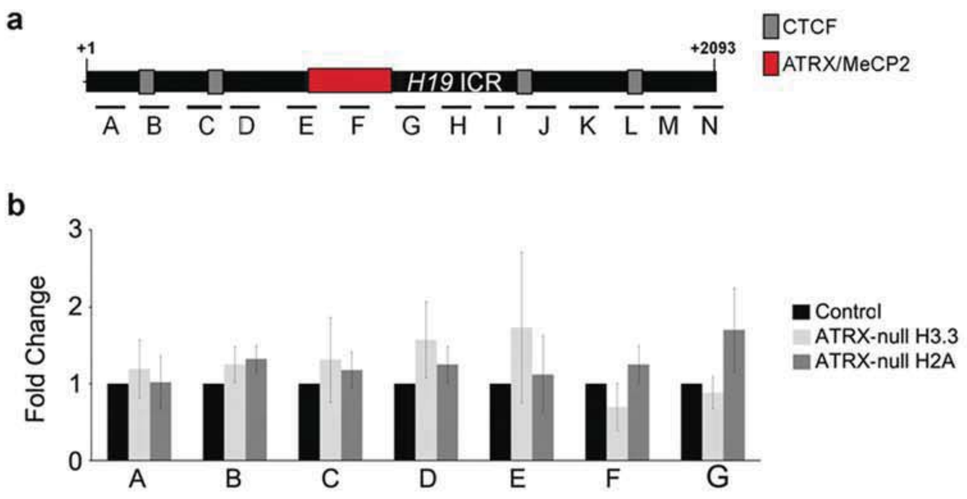

Table 1. Common 4C interaction sites of the *H19* ICR, genomic location and nearest gene.

Chromosome	Restriction Fragment		Location	Closest Gene
**chr1**	26858209	26859582	Intergenic	*4931408C20Rik*
**chr2**	98499779	98512366	Genic	*Chl1*
**chr3**	73410459	73413706	Intergenic	*Glrx3*
**chr3**	108870234	108874288	Intergenic	*Dusp8*
**chr6**	73330248	73337200	Intergenic	*Dusp8*
**chr6**	103597525	103600507	Intergenic	*GM459*
**chr7**	144866094	144872574	Intergenic	*GM459*
**chr7**	149261169	149263375	Intergenic	*Krtap5-4*
**chr7**	149307668	149313341	Genic/Intergenic	*Ifitm10*
**chr7**	149438230	149439113	Genic	*Ifitm10*
**chr7**	149439113	149447176	Genic	*Ifitm10*
**chr7**	149498852	149500413	Genic	*Ifitm10*
**chr7**	149509326	149513253	Genic	*Ifitm10*
**chr7**	149513253	149515356	Intergenic	*Ctsd*
**chr7**	149515356	149519553	Intergenic	*Syt8*
**chr7**	149519553	149525721	Intergenic	*Lsp1*
**chr7**	149527044	149540622	Genic	*Lsp1*
**chr7**	149595286	149597921	Genic	*Lsp1*
**chr7**	149610253	149618357	Intergenic	*Mrpl23*
**chr7**	149644830	149646405	Intergenic/Genic	*Mrpl23*
**chr7**	149646405	149655084	Intergenic/Genic	*Mrpl23*
**chr7**	149655111	149664996	Intergenic/Genic	*Nctc1*
**chr7**	149711578	149716624	Intergenic/Genic	*Nctc1*
**chr7**	149716624	149721297	Intergenic	*Nctc1*
**chr7**	149721297	149728697	Intergenic/Genic	*H19*
**chr7**	149728697	149733529	Intergenic	*H19*
**chr7**	149733529	149746257	Intergenic	*H19*
**chr7**	149746257	149753414	Intergenic	*H19*
**chr7**	149753414	149764107	Intergenic	*H19*
**chr7**	149764107	149767890	Intergenic	*Igf2*
**chr7**	149767890	149768026	Genic	*R74862*
**chr7**	149768026	149775715	Intergenic	*Mcm5*
**chr7**	149778665	149782077	Intergenic	*4930433N12Rik*
**chr7**	149826877	149830434	Intergenic	*4933422A05Rik*
**chr7**	150224953	150227490	Intergenic	*2310057J18Rik*
**chr8**	77666553	77671125	Intergenic	*Vstm2a*
**chr9**	0	3043146	Intergenic	*1700012B15Rik*
**chr9**	35107267	35115588	Genic	*AK0152361/GM2897/GM5795/1700110101Rik*
**chr10**	28733339	28739549	Intergenic	*Lrrc4c*
**chr11**	15963427	15968209	Intergenic	*GM20754*
**chr12**	3105261	3110529	Intergenic	*4930443G12Rik*
**chrX**	120113459	120128574	Intergenic	*3110007F17Rik*
**chrX**	120775611	120790133	Intergenic	*Vmn2r121*
**chrX**	121290652	121305516	Intergenic	*Vmn2r121*
**chrY**	2785883	2794230	Intergenic	*Rbmy*
**chrY**	2855890	2861204	Intergenic	*Rbmy*
**chrY**	2861896	2870363	Intergenic	*Rbmy*
**chrY**	2877622	2893659	Intergenic	*Rbmy*

Table 2. Common 4C interaction sites of the *Gtl2* DMR, genomic location and nearest gene.

Chromosome	Restriction Fragment		Location	Closest Gene
**chr2**	27351686	-27361852	Genic	*Brd3*
**chr2**	98499779	-98512366	Intergenic	*Lrr4c*
**chr7**	149764107	-149767890	Intergenic	*H19*
**chr8**	19922549	-19927181	Intergenic	AK166824
**chr8**	19932555	-19943252	Intergenic	*GM15319*
**chr9**	0	-3043146	Intergenic	*DQ702609*
**chr11**	3017512	-3033801	Genic/Intergenic	*Pisd-ps1*
**chr11**	3033801	-3041172	Genic	*Pisd-ps1 and Sfi1*
**chr11**	3094101	-3100093	Intergenic	*Sfi1*
**chr11**	59240597	-59242772	Genic	*Snap47*
**chr11**	75934667	-75944365	Genic	*Vps53 and Glod4*
**chr12**	3105261	-3110529	Intergenic	1700012B15Rik
**chr12**	73112304	-73113296	Intergenic	*Daam1*
**chr12**	110159632	-110160109	Genic	*Wdr25*
**chr12**	110168065	-110190282	Genic	*Wdr25*
**chr12**	110238956	-110254990	Genic	*Wdr25*
**chr12**	110588480	-110588909	Intergenic	*Gtl2/Dlk1*
**chr12**	110588909	-110590456	Intergenic	*Gtl2/Dlk1*
**chr12**	110656819	-110657727	Intergenic	*Gtl2/Dlk1*
**chr12**	110657727	-110666346	Intergenic	*Gtl2/Dlk1*
**chr12**	110718842	-110722099	Intergenic	*Gtl2/Dlk1*
**chr12**	110756878	-110761114	Intergenic	*Gtl2/Dlk1*
**chr12**	110761114	-110762939	Intergenic	*Gtl2/Dlk1*
**chr12**	110762939	-110766876	Intergenic	*Gtl2/Dlk1*
**chr12**	110766876	-110771307	Intergenic	*Gtl2/Dlk1*
**chr12**	110771307	-110773703	Intergenic	*Gtl2/Dlk1*
**chr12**	110773703	-110783827	Intergenic	*Gtl2/Dlk1*
**chr12**	110783827	-110787839	Intergenic	*Gtl2/Dlk1*
**chr12**	110808671	-110812154	Intergenic	*Gtl2/Dlk1*
**chr12**	110812154	-110813737	Intergenic	*Gtl2/Dlk1*
**chr12**	110836641	-110842682	Intergenic	*Gtl2/Dlk1*
**chr12**	110842682	-110855989	Intergenic	*Gtl2/Dlk1*
**chr12**	110855989	-110861560	Intergenic	*Gtl2/Dlk1*
**chr12**	110869303	-110875353	Intergenic	*Gtl2/Dlk1*
**chr12**	110932677	-110935827	Intergenic	*Gtl2/Dlk1*
**chr12**	110935827	-110942651	Intergenic	*Gtl2/Dlk1*
**chr12**	111288801	-111289110	Genic	*Ppp2r5c*
**chr12**	115782235	-115788556	Genic	*abParts*
**chr14**	79426258	-79428915	Intergenic	*1300010F03Rik*
**chr17**	27428406	-27450273	Intergenic	*AK038843*
**chr17**	36614864	-36625205	Intergenic	*H2-M10.4*
**chr18**	19071557	-19075317	Intergenic	*Dsc3*
**chrX**	53396963	-53414549	Genic/Intergenic	3830403N18Rik
**chrX**	120113459	-120128574	Intergenic	3110007F17Rik
**chrX**	120322524	-120336463	Intergenic	3110007F17Rik
**chrX**	120775611	-120790133	Intergenic	3110007F17Rik
**chrX**	121002628	-121017144	Intergenic	3110007F17Rik
**chrX**	121290652	-121305516	Intergenic	*Vmn2r121*
**chrX**	121305516	-121308352	Intergenic	*Vmn2r121*
**chrX**	121511155	-121525638	Intergenic	*Vmn2r121*
**chrX**	121762997	-121777512	Intergenic	*Vmn2r121*
**chrX**	121939601	-121954064	Intergenic	*Vmn2r121*

**chrX**	122202281	-122216780	Intergenic	*Vmn2r121*
**chrX**	122331634	-122346125	Intergenic	*Vmn2r121*
**chrX**	166419401	-166516970	Genic/Intergenic	*Mid1*
**chrY**	1480810	-1486269	Intergenic	*Zfy2*
**chrY**	2780466	-2783962	Intergenic	*Rbmy1a1*
**chrY**	2785883	-2794230	Intergenic	*Rbmy1a1*
**chrY**	2794230	-2848781	Intergenic	*Rbmy1a1*
**chrY**	2848788	-2854570	Intergenic	*Rbmy1a1*
**chrY**	2855890	-2861204	Intergenic	*Rbmy1a1*
**chrY**	2861896	-2870363	Intergenic	*Rbmy1a1*
**chrY**	2870363	-2872247	Intergenic	*Rbmy1a1*
**chrY**	2874492	-2877622	Intergenic	*Rbmy1a1*
**chrY**	2877622	-2893659	Intergenic	*Rbmy1a1*
**chrY**	2894176	-2897414	Intergenic	*Rbmy1a1*

SUPPLEMENTARY FIGURE LEGENDS

Supplementary Figure 1. ATRX is not required for transient proximity of the *H19* ICR and other imprinted domains in neocortical cells (a) Venn diagrams show the number of 1Mb regions which are commonly enriched in both 4C-seq biological replicates. Interactions of the *H19* ICR *in trans* are represented on the left and interactions *in cis* are represented on the right. (b) Analysis of genomic distribution of *H19* ICR interacting fragments on each chromosome reveals that the majority of reproducible interactions occur within chromosome 7 while *trans* interactions are distributed across the genome. (c) 3D FISH of mouse neocortical sections using BAC probes of various imprinted domains. Representative collapsed confocal series (Z-Series) and 3D-reconstructed serial confocal images (3D View) of neonatal cortical nuclei showing superimposed FISH signals for BACs corresponding to *H19/Igf2* (green) and other IGN members (red). Sections were counterstained with DAPI (blue). Scale bar: 0.5 μm. (d) Frequency of hybridization signals of imprinted domains that are either overlapping or in close physical proximity to *H19/Igf2* DNA FISH signal. Graphed data represent the mean interaction frequency from three control and ATRX-null littermate pairs (n=100 nuclei analyzed for each animal). Signals with a 3D centre-to-centre distance of less than 1 μm were considered to represent an interaction. Using these criteria, *Slc38a4*, *Grb10*, *Dlk1/Gtl2*, *Dcn*, *Zac1* and *Mest* are sometimes found in spatial proximity to *H19/Igf2*, while *Peg10/Sgce* and *Gapdh* do not co-localize with *H19/Igf2*. Interaction frequencies were not affected in the ATRX-null forebrain.

Supplementary Figure 2. Liver and forebrain 3C analysis. (a) Schematic representation of the *H19/Igf2* imprinted domain and 3C assay as depicted in Figure 1. (b) 3C chromatin looping analysis confirms previous interactions reported in the neonatal liver (Qiu et al. 2008), and identifies a similar interaction profile in the forebrain. Analysis with a negative bait sequence (-100 kb) does not produce any significant interactions. (n=3, error bars depict SEM) (c) Example of a 3C interaction profile from one control/ ATRX-null pair and (d) corresponding quantitative RT-PCR analysis of H19 and Igf2 expression. Error bars represent standard deviation of technical error.

Supplementary Figure 3. H3.3 ChIP shows that levels of this histone variant are not altered at the H19 ICR in the ATRX-null brain. **(**a) Schematic of the *H19* ICR and alignment of primers. (b) ChIP in neonatal control and ATRX-null brains demonstrates a modest increase in H3.3 and H2A ChIP signal within the 5' region of the *H19* ICR.
